# A Systematic Review of the Criminogenic Potential of Synthetic Biology and Routes to Future Crime Prevention

**DOI:** 10.3389/fbioe.2020.571672

**Published:** 2020-10-06

**Authors:** Mariam Elgabry, Darren Nesbeth, Shane D. Johnson

**Affiliations:** ^1^Dawes Center for Future Crime, Jill Dando Institute, Department of Security and Crime Science, University College London, London, United Kingdom; ^2^Advanced Centre for Biochemical Engineering, University College London, London, United Kingdom

**Keywords:** biocrime, cyberbiosecurity, synthetic biology, systematic review, crime science, crime harvest, biosecurity

## Abstract

Synthetic biology has the potential to positively transform society in many application areas, including medicine. In common with all revolutionary new technologies, synthetic biology can also enable crime. Like cybercrime, that emerged following the advent of the internet, biocrime can have a significant effect on society, but may also impact on peoples' health. For example, the scale of harm caused by the SARS-CoV-2 pandemic illustrates the potential impact of future biocrime and highlights the need for prevention strategies. Systematic evidence quantifying the crime opportunities posed by synthetic biology has to date been very limited. Here, we systematically reviewed forms of crime that could be facilitated by synthetic biology with a view to informing their prevention. A total of 794 articles from four databases were extracted and a three-step screening phase resulted in 15 studies that met our threshold criterion for thematic synthesis. Within those studies, 13 exploits were identified. Of these, 46% were dependent on technologies characteristic of synthetic biology. Eight potential crime types emerged from the studies: bio-discrimination, cyber-biocrime, bio-malware, biohacking, at-home drug manufacturing, illegal gene editing, genetic blackmail, and neuro-hacking. 14 offender types were identified. For the most commonly identified offenders (>3 mentions) 40% were outsider threats. These observations suggest that synthetic biology presents substantial new offending opportunities. Moreover, that more effective engagement, such as ethical hacking, is needed now to prevent a crime harvest from developing in the future. A framework to address the synthetic biology crime landscape is proposed.

## Introduction

Synthetic biology empowers us with the ability to re-program living organisms to produce useful products and processes, including medicinal ones that can improve our quality of life. When considering synthetic biology as an engineering science (Andrianantoandro et al., 2006), the introduction of the capability to “program” a biological system can be compared to the introduction of the internet and the capability of programming a computer. A traditional biological system could, for example, be modified to behave like a sensor that gets activated and emits a signal in the presence of a toxin or disease signature, which is useful for medical diagnostics or environmental solutions (Bhutkar, 2005). Following this analogy, the introduction of the internet brought palpable benefits to society, increasing connectivity, expanding commerce and increasing knowledge sharing around the world. While the legitimate benefits of the internet are clear, misuses soon emerged as offenders identified and exploited the crime opportunities that cyberspace offers. The exploitation of these opportunities has been so significant that around half of all crime is now committed online (2017). This occurred as the internet was not designed with security in mind. Unfortunately, inattention to the crime and security implications of new technologies (and services) is common, with a contemporary example being the security vulnerabilities associated with consumer internet connected devices (Alladi et al., 2020). Synthetic biology integrates a diverse set of technologies to enable various applications that have enormous potential. While these were once restricted to specialized institutions, they are now freely available online through kits, bioinformatics tools and data (Serrano, 2007), increasing the reach (and reducing the cost) of these technologies for legitimate purposes. However, unless adequate security is designed in, the availability of these technologies has the potential to increase the opportunity for their misuse, such as the “home-brewing” of recreational drugs (Endy et al., 2015) or generating peoples' portraits from discarded DNA samples (Dewey-Hagborg, 2013). In fact, a 2019 trend report published by the UK Home Office highlighted designer psychoactive drugs and synthetic biology by amateurs as future security concerns (UK Home Office, 2019). Misuse is traditionally defined as illegitimate activities that are punishable by law. However, the exploitation of legitimate activities for “criminal” purposes—for which policies do not yet exist—must also be taken into account as new opportunities for crime surface with new technologies, increasing as they mature (Pease, 1997). While the misuse of the internet may have so far largely been limited to the digital space (for an exception, see Lee et al., 2016), the misuse of synthetic biology could have direct effects on an individual's or the public's health. To take an example of such potential effects, consider the debate of published research reporting directed evolution experiments conducted in 2012 of the deadly bird-flu virus H5N1 to study influenza transmission in humans (Imai et al., 2012). Forecasting future crime opportunities that may be facilitated by emerging technology, such as synthetic biology, permits for preventative steps to be put in place ahead of time, to safeguard against potential misuses.

Biocrime is here defined as the exploitation of vulnerabilities in biological tools, data/databases, devices, or techniques for criminal purposes and can be either categorically new, or a combination of current crime types. It is enabled by both the increase in biological data created and the decreasing costs of the technology used. Sequencing the human genome in the 2000's for example cost $100 million and required highly specialized institutions and expertise (Wetterstrand, 2020). Today this is possible for just under $1000, with emerging companies offering $200 kits that can be purchased for use “at-home” (Molteni, 2018). Furthermore, while increasing amounts of biological data are being produced and digitized for legitimate purposes, these same types of data are also being sold on the black market—at 10–20 times more per record than, for example, credit card data (Backes et al., 2016; Czeschik, 2018). This biological data includes genetic information that is collected and exchanged for monetary gain. An example is the data leakage scandal whereby commercial DNA testing kit provider 23AndMe sold thousands of customers personal data to GlaxoSmithKline for $300 million (Brodwin, 2018) without customer informed consent. As is the data breach of diagnostic and genetic test provider LifeLabs affecting 15 million Canadians (Abedi, 2019). Unlike financial information or other types of data, biological information cannot be arbitrarily changed, which makes it more vulnerable. For example, if compromised, bank details can be revoked which prevents offenders from exploiting them. Since biological data cannot be changed in the same way, once stolen, it is difficult to prevent its exploitation (e.g., its use in ransoms). Two recent examples which illustrate the point are the attacks on the U.K. National Health Service (NHS) and Eurofins (Luxembourg), a major commercial provider of forensic processing services for the U.K. These systems were infiltrated by a computer “virus” through an innocuous email opened by staff that spread through vulnerable computer systems, encrypting data, and locking out users until a ransom was paid. These attacks were estimated to have cost £92 million (Field, 2018) and €4 million−163 million (Alpha Value, 2019), respectively. Moreover, there were additional losses that could not be costed. For example, during the NHS WannaCry attack, medical professionals could not order tests, view results, track patients or type in notes, leading to the cancellation of scheduled surgeries as the electronic, imaging and drug-prescribing systems were frozen (Clarke and Youngstein, 2017). The attack on Eurofins Laboratory created a backlog of almost 20,000 cases under investigation that required evidence for DNA in samples found at crime scenes (Muncaster, 2019). With a U.K. bio-economy plan for 2030 worth £220 billion, bio-crime prevention becomes unquestionably important.

Current measures intended to combat biocrime are incomplete, as they are limited to the use of biological agents in isolation and do not consider vulnerabilities in today's extended supply chain. Today, biotechnology comprises integrated workflows that increasingly depend on computer-controlled and automated systems. This creates efficiencies but also new opportunities for biocrime. Consequently, as noted above, biocrime is conceptualized here to include offenses that involve biological and cyber systems to commit categorically new offenses, traditional offenses, or some combination of the two. Forms of biocrime are considered here from a crime opportunity perspective, in particular, the routine activity approach (Felson and Cohen, 1980). We take this approach as it provides a framework for thinking about what and who might influence the likelihood of a crime event. According to Felson and Cohen (1980), for a crime to occur, a motivated offender and suitable target need to converge in space (physical and/or virtual) and time in an unguarded place (Llinares and Johnson, 2018; Wachs et al., 2020). Absent this convergence, crime is unlikely or even impossible. Each element (motivated offender, suitable target, and unguarded place) has a “controller” that can influence these interactions locally (e.g., a place manager and the policies adopted), which are in turn influenced by “super-controllers” (e.g., governments and internationally agencies) who have an influence on (say) place managers and hence influence “chemistry for crime” more indirectly (Sampson et al., 2010). Considering the role of each of these actors is thus useful in the context of preventing new or emerging crimes (hereafter crime harvests) (Pease, 2002) since each can influence the likelihood of crime in different ways.

Although the current rate of incidents of biocrime may be low these risks should not be ignored as their impacts can be severe. The ongoing challenge of the severe acute respiratory syndrome coronavirus 2 (SARS-CoV-2) pandemic reveals weaknesses in our healthcare system, our biosecurity protocols and our preparedness to act. An example being the U.K.'s hampered effort to increase its testing capacity by outsourcing kits from Eurofins Laboratory (Luxembourg). A great disappointment, orders had been found to be contaminated with SARS-CoV-2, delaying the availability of these kits to the public and identifying major issues in the international supply chain (Gardner and Yorke, 2020). While there is no suggestion here that the pandemic or the contamination of testing kits involved criminal intent, it highlights how easily biotechnology supply chains can be compromised unintentionally. Following the same theme, perhaps more worryingly, researchers were able to reconstruct chemically synthesized clones of the SARS-CoV-2 virus within a week of receiving synthetic DNA fragments (Thi Nhu Thao et al., 2020). However, while SARS-CoV-2 provides a timely example for motivating the work reported here, it is an extreme example that fails to convey the range of offenses that may be possible and that we should be seeking to prevent.

As cyber-physical spaces in biotechnology become more integrated, the attack surface for biocrime expands to create opportunities for data exploitation, but also the manipulation and misuse of biological material. Moreover, developments in the synthetic biology industry may facilitate the commission of crime across geographic boundaries as laboratories become more automated and internet-connected (Peccoud et al., 2018). Synthetic biology-enabled crime types will likely require multidisciplinary expertise to detect and prevent, involving collaborations with (for example) computer scientists, bioinformaticians, molecular biologists, and information technologists (Murch et al., 2018; Richardson et al., 2019). As a result and according to Richardson et al. (2019), “*as more connections between traditionally isolated systems are developed, more security controls must be considered in order to mitigate risks and reduce vulnerabilities*,” requiring the generation of a “*full stack biotechnologist*” (Lewis, 2019) that combines skills from the Life sciences (such as DNA technologies, bioprocessing) with those traditionally considered Computer science skills (such as computer programming, machine learning, cybersecurity) (Faezi et al., 2019). Because the stakes are high, it is important that the risks associated with biocrime are assessed, and preparations made to prevent them are set in motion sooner rather than later.

Cyberbiosecurity is emerging as a field of enquiry (Peccoud et al., 2018) that addresses the changing threat of biocrime. To inform this effort we conduct a Systematic Review (SR) to synthesize the knowns and unknowns in current and predicted biocrime. While *ad-hoc* literature reviews can produce a patchy and biased coverage of the literature, SRs are designed to minimize bias by using systematic and transparent search strategies to extract as much of the available evidence as possible on a particular topic (Cockbain et al., 2018). As the topic of biocrime is emerging, the scale of knowledge and gaps in it are currently unknown. Hence, performing a SR would provide a clearer picture of the current state of research. Typically, SRs are conducted to synthesize evidence on “what works” in fields such as medicine (Curtis and Cairncross, 2003) where data are plentiful. However, SRs are also useful where data are more fragmented and for emerging issues, such as new crime trends, where they can help summarize the state-of-the-art, assess the quality of the existing research, identify gaps in knowledge and encourage further work, where needed (Blythe and Johnson, 2019). Here, four objectives were pursued, to synthesize the evidence on:

1. What forms of biotechnology are criminally exploitable? (Section Four Criminally Exploitable Biotechnologies Identified)
2. What crime types are facilitated? (Section Thirteen Exploits Identified)
3. What are the influencing factors for a crime to occur? (Section Five Crime Influencing Factors)
4. What interventions can be put in place now to prevent their occurrence in the future? (Section Discussion)

## Methods

### Study Design (SR Protocol)

**Figure 1:**
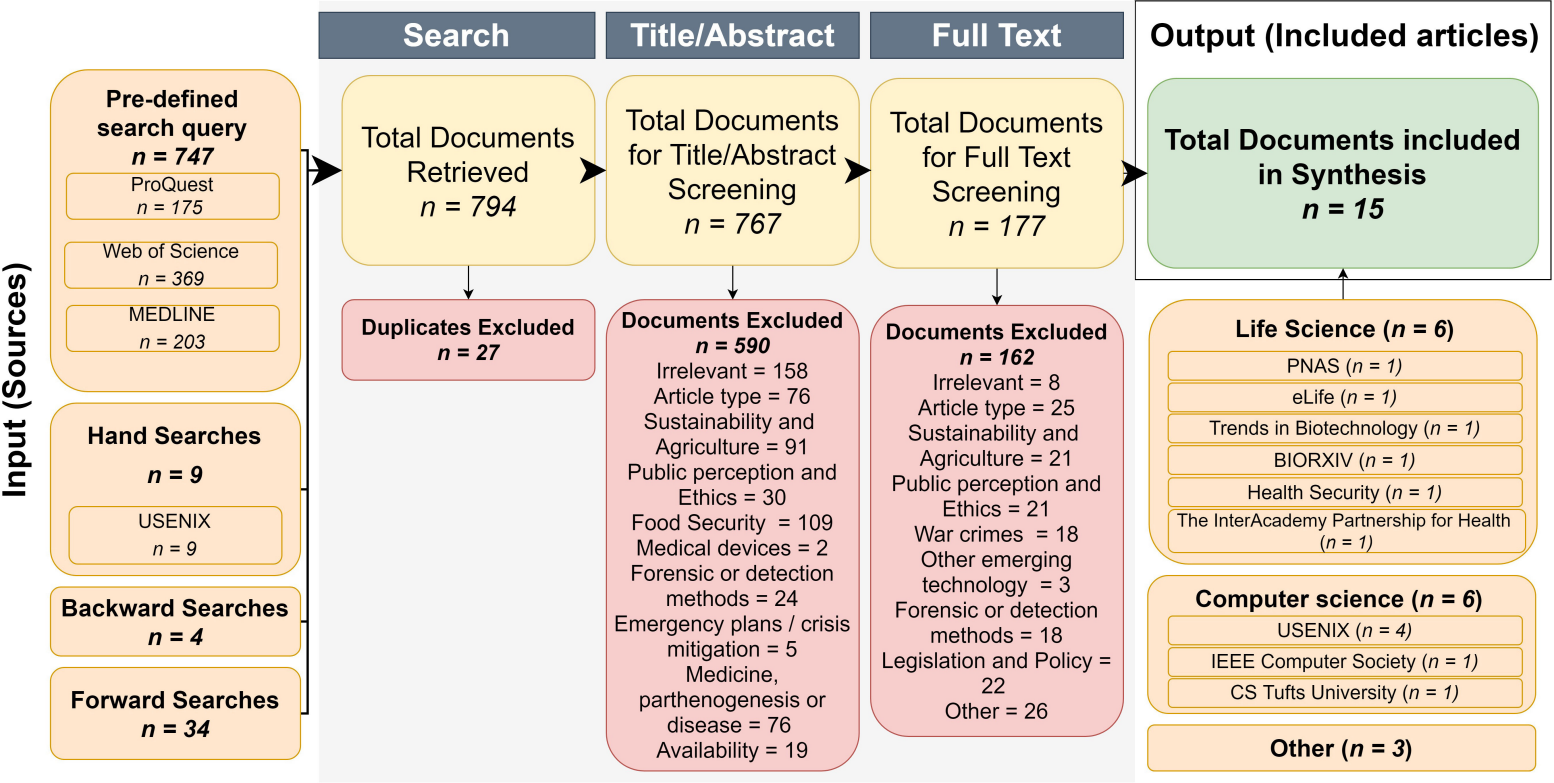
Flow chart of the three-step article identification procedure implemented. We sought to identify peer-reviewed articles that considered exploitable biotechnologies using a pre-defined search query and a set of eligibility criteria (organized according to the PICOS format, see Table 1) for articles by querying four databases Medline, Web of Science, ProQuest, and USENIX. A curation procedure was then applied to the initial 794 hits (first column in diagram, entitled “Input Sources”) that consisted of three steps: Search, Title/Abstract, and Full Text (gray boxes). The Search step excluded duplicate studies. The Title/Abstract step excluded articles that did not meet the inclusion criteria (see Table 1) in their Title and Abstract. The Full-text step excluded articles on the basis of relevancy (inclusion criteria) based on their full text. Only the final 15 articles (final column in diagram, entitled “Output Included articles” in the green box) were considered for the thematic synthesis described in this study.

We previously devised a SR protocol that adhered to the Preferred Reporting Items for Systematic Review and Meta-analysis Protocols (PRISMA-P) guidelines. The SR protocol is registered with PROSPERO (CRD42019131685) and was peer-reviewed and published in Elgabry et al. (2020). The SR process and results that arose from applying this SR protocol are depicted in Figure 1.

The first stage involved the use of a pre-defined and optimized search query to identify relevant articles. This was conducted between April and August 2019. We searched three electronic databases (ProQuest, Web of Science, and MEDLINE) (Figure 1) using a keyword search which employed combinations of the following terms; genetic engineering, synthetic biology, biotechnology, threat(s), threatening, crime(s), criminal(s), criminogenic, offend, offender(s), offending, secure, securing, security, hack(s), hacking, hacker(s). Additionally, the USENIX (Advanced Computing Systems Association) database was hand searched as it did not have Boolean search functions for the retrieval of papers. The U.K. Government website and Global dissertations database were also searched to identify relevant “gray” literature. We conducted backward and forward searches to further identify relevant publications. Forward searches involve finding (additional) studies that have cited key studies, while backward searches involve the identification of relevant past works that were cited in the reference lists of already identified articles (Greenhalgh and Peacock, 2005; Zhang et al., 2011).

**Table 1:** A summary of the eligibility criteria for the screening phases of the systematic review.

**Criteria**	**Inclusion**	**Exclusion**
**Population(s)**	**Human**	**Animal, plant**
Intervention(s)	Current or potential future misuse of biotechnology, synthetic biology, and genetic engineering	Technology: medical devices Crime types: war crimes, crimes against humanity, intellectual Property and corporate liability crimes, agriculture and food security, wildlife/biodiversity crimes
Comparator	Not applicable	Not applicable
Outcomes	Criminally exploitable biotechnology Crime types and sub-types Individual/system-level characteristics of population/sector involved	The crime themes extracted are synthesized for implications in the U.K. only.
Study design	Peer-reviewed, government document, or academic thesis only All study designs are included that explicitly discuss or demonstrate an attack model.	Commentaries Forewords Books/book reviews Articles Opinions Letters Editorials
Other	English language	Non-english

*Reproduced from: Elgabry et al. (2020)*.

### Search Strategy and Eligibility Criteria

The second and third stages of the SR process (Figure 1) involved screening articles against pre-set inclusion criteria (see Table 1) organized according to the PICOS format (Richardson et al., 1995; Sackett et al., 1997; Schardt et al., 2007), as follows. All articles written in English were considered, regardless of the year of publication. Only articles with a study design were included, which means opinions and commentaries were excluded. The only exception to this rule was review articles. Only studies that explicitly made a link between biotechnology, synthetic biology, or genetic engineering and technological misuse (by discussing or demonstrating threat/attack models) were included (Table 1, Figure 1). Studies that focused exclusively on medical devices or war crimes, crimes against humanity, intellectual property and corporate liability crimes, agriculture and food security, wildlife or biodiversity crimes were excluded (Table 1, Figure 1). Studies that discussed policy but without any discussion of the underlying technology or criminal misuses were also excluded. Some of the included studies discussed multiple crime types; any categorized out of the scope of the paper were removed.

### Data Extraction and Synthesis

Using the PICOS criteria, studies were first screened on the basis of their titles and abstracts and then on the basis of the full text. All articles were screened by the lead author, and a random sample (20%) were screened by the co-authors at each stage of the review process. This allowed us to assess inter-rater reliability and coder drift (Byrt et al., 1993) by calculating the PABAK statistic as per Smith et al., 2011. In the current study, the PABAK scores of 0.95 and 0.8 at the Title/Abstract and Full-Text stages (respectively) indicated near-perfect agreement between reviewers.

All articles were managed and coded using the online tool Eppi Center Reviewer software (Thomas et al., 2010). For each of the articles ultimately reviewed (Figure 1), the following variables were collected (Supplementary Table 1):

- Study identifiers: country, date published, study discipline,
- Study design: speculative, experimental or “currently occurring in the wild,”
- Biotechnology,
- Exploit and Crime type,
- Offender threat model
- Main findings reported.

A thematic qualitative approach was taken to synthesize and identify the crime type themes in the literature (Thomas and Harden, 2008). Studies were also assessed according to the extent to which they could be said to be plausible, given the study design employed. For example, findings from studies in which a proof-of-concept was demonstrated experimentally in a laboratory setting were considered more plausible than those from studies where a crime was discussed as possible by researchers but for which there was no actual test of the attack model. Three types of study design, “speculative,” “experimental,” and “currently occurring in the wild,” were considered, based on an *a-priori* hierarchy of evidence (Blythe and Johnson, 2019). “Speculative” study designs employed a survey approach involving qualitative methods, such as the Delphi technique (Hsu and Sandford, 2007), for which experts are asked to generate and collectively rate scenarios. “Experimental” study designs involved demonstrations of a scenario or the identification of vulnerabilities in a system using (for example) penetration testing, whereby a system is examined through a detailed analysis of potential threats and attack models (Bishop, 2007). Finally, articles that described crime types as “currently occurring in the wild” were those that discussed reports of actual misuse in the real-world.

## Results

Figure 1 shows the number of articles considered at each stage of the review. Initial searches yielded a total of 794 records. After the removal of 27 duplicates, there was a total 767 unique publications. Of these, 177 met the criteria for full-text screening, and of these, 15 met the eligibility criteria and were included for synthesis. Eighty percent of all articles were published in either the Life (6/15) or Computer sciences (6/15). Of the publications from Computer Science, half were published in USENIX. The remaining three papers were published in different disciplines: policy research by the private Smith Richardson Foundation, Foresight studies, and a master's dissertation for Security studies from the American Naval postgraduate school.

**Figure 2:**
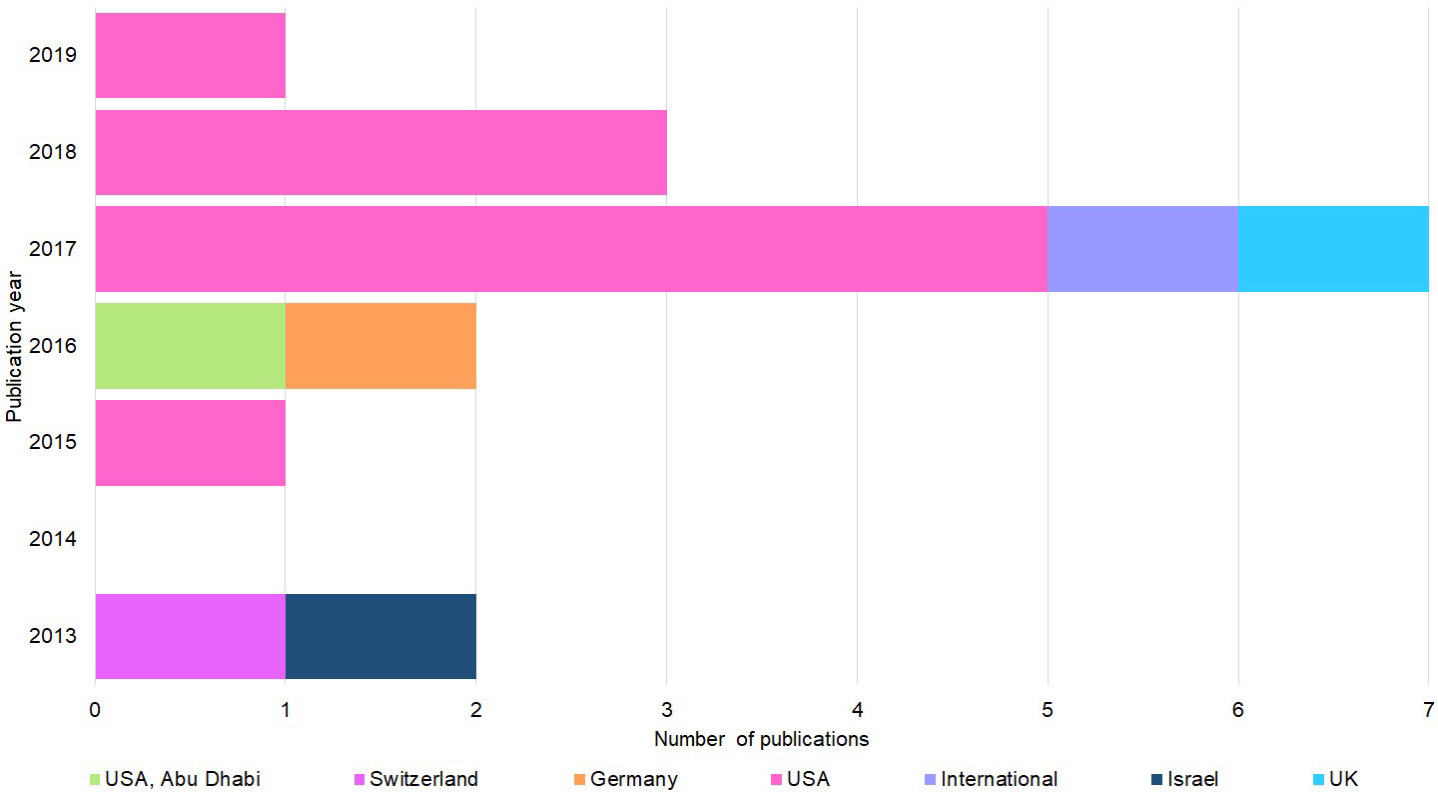
Publication year and country origin of the 15 screened studies. Nation of origin breakdown and publication date of the 15 studies (Figure 1) considered for thematic synthesis.

Figure 2 shows that the 15 studies were published from 2013 onwards and approaching half (6/15) were published in 2017. Most papers (9/15) were written by U.S. authors, with only one paper written by authors from the U.K., Israel, Switzerland, or Germany. Finally, only two papers involved an international collaboration.

The included articles varied in terms of their study design (Table 2). Six articles used an experimental design, all in the form of penetration testing. In contrast, nine were speculative in nature and used a mixture of horizon scanning and scenario building approaches, with insights elicited from experts. None of the articles reported crimes that could be described as “currently occurring in the wild.”

**Table 2:** A summary of the crime types that emerged.

**Study method**	**Study design (speculative, experimental, “currently occurring in the wild”)**	**Authors, year**	**Exploit (13 total)**		**Domain (bio-related, cyber-related, drug-related)**	**Crime type**	**Estimated timescale**
Penetration testing	Experimental	Ayday et al. (2013)	1	Privacy breaches of genomic data within a clinical setting	Cybercrime	Bio-discrimination	Current^*^
2-7	Experimental	Backes et al. (2016)	2	Privacy breaches through the identification and matching of Epigenetic data in both a clinical setting and for the biomedical research community	Cybercrime	Bio-discrimination	
2-7	Experimental	Ney et al. (2017)	3	Sequencing physical DNA malware that compromises the computer that processes it	Biocrime	Bio-malware	
2-7	Experimental	Faezi et al. (2019)	4	Confidentiality breach and oligonucleotide sequence theft through an acoustic side channel attack	Cybercrime	“Cyber-biocrime”	
2-7	Experimental	Franzosa et al. (2015)	5	Privacy breach of human microbiome data within a research setting	Cybercrime	Bio-discrimination	
2-7	Experimental	Ney et al. (2018)	6	Tampering with DNA sequencing machines to modify sequencing results (enabling “Targeted mis-genotyping”)	Biocrime	“Cyber-biocrime”	
1-8 Expert workshop	Speculative	Fears and ter Meulen (2018)	7	Exploitation of genome editing technology such as CRISPR to engineer the human microbiome, immune system or to make illegal changes to the inherited genome.	Biocrime	Illegal gene editing and “neuro-hacking”	Long-term
2-8	Speculative	Kirkpatrick et al. (2018)	–	Exploitation of genome editing technology such as CRISPR to engineer the human microbiome, immune system or to make illegal changes to the inherited genome.	Biocrime	Biohacking, illegal gene editing and “neuro-hacking”	Not discussed
1-8 Horizon scanning and delphi	Speculative	Hauptman and Sharan (2013)	8	Exploitation of synthetic biology technologies. For example, the DNA of human individuals is misused for extortion	Cybercrime	“Genetic blackmail”	2016–2025
1-8 Horizon scanning and delphi and scenario building	Speculative	Wintle et al. (2017)	–	Exploitation of synthetic biology technologies to engineer microorganisms for illegal purposes (e.g., engineer bacteria to cause infection).	Biocrime	“Cyber-biocrime,” DIY drugs, biohacking, illegal gene editing and “Genetic blackmail”	5–10 years
1-8 Scenario building	Speculative	Bress (2017)	9	Illegal use and manufacturing of drugs using emerging technology	Drug-related	DIY drug manufacturing and biohacking	2030
1-8 Author speculation	Speculative	Dieuliis and Giordano (2017)	10	Exploitation of the microbiome to gain indirect control of the brain.	Biocrime	“Neuro-hacking”	Not discussed
2-8		Ali et al. (2016)	11	Hacking cyberinfrastructure (e.g., supply chain) of Digital Microfluidic Biochips (DMFB) to compromise assay outcomes, leak sensitive information, or damage the DMFB making it unusable.	Cybercrime	“Cyber-biocrime”	<5 years ^*^
Literature review	Speculative	Peccoud et al. (2018)	12	Hacking cyberinfrastructure of integrated biotechnology workflows (e.g., biomanufacturing processes) to compromise operations and/or produce nefarious products.	Cybercrime	“Cyber-biocrime”	Current^*^
2-7	Speculative	Qu (2019)	13	Exploitation of genomic information through data breaches to engage in blackmail and/or privacy breaches.	Cybercrime	Bio-discrimination	

Content analysis of the extracted data (Supplementary Table 1) from the 15 reviewed studies (Figure 1) is summarized and ordered according to study design, with experimental study designs at the top. Studies are labeled by study design to signify articles that are either speculative, experimental, or “currently occurring in the wild.” Articles with experimental designs demonstrate feasibility of the method used to commit the exploit whilst speculative articles discussed a potential exploit without a proof of concept. Articles that discussed reports of actual misuse in the real-world are “currently occurring in the wild,” Thirteen exploits were extracted from the 15 reviewed studies and divided into 3 domains: bio-related, cyber-related, and drug-related crime. The total 13 exploits were also grouped to generate the overarching crime types (emerging crime opportunities facilitated by the biotechnologies) (Figure 5). Where available, the estimated timescale predicted by the authors or participants of a study is provided.

* *= timescale not explicitly stated by the authors of the paper but implied (by demonstrating a proof-of-concept through penetration testing, for example) and therefore interpreted/estimated by the researcher*.

### Four Criminally Exploitable Biotechnologies Identified

**Figure 3:**
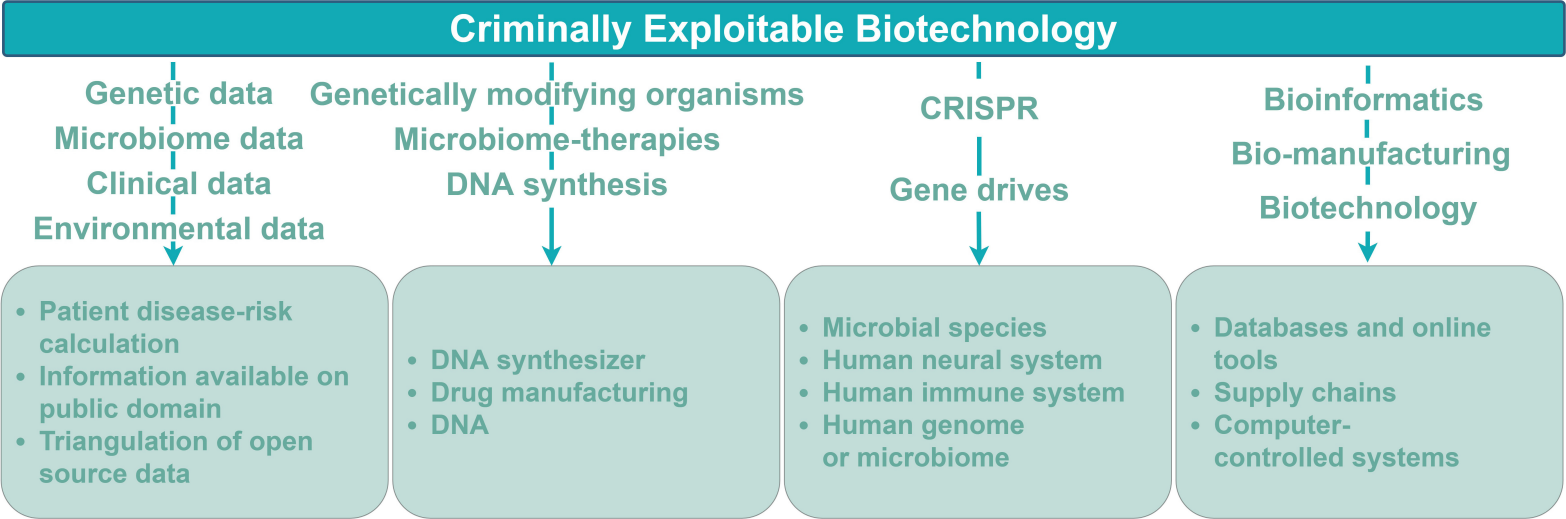
A typology of criminally exploitable biotechnology. The 15 studies (Figure 1) were analyzed with respect to the criminally exploitable biotechnology each study identified. Across the 15 studies, the discussed risks predominantly related to technologies characteristic of synthetic biology research. The text over a downward pointing arrow lists the biotechnology or data exploited. The bottom row of boxes summarizes the means by which the biotechnology may be exploited.

Figure 3 summarizes the types of biotechnologies or data discussed across the articles and the crime risks identified. The former are summarized in this section, the latter in the next. The 15 reviewed articles predominantly discussed risks relating to technologies characteristic of synthetic biology research. Across the 15 articles, 20% (3/15) identified criminally exploitable risks related to the storage, handling, or processing of biological data such as genetic, microbiome, epigenetic, environmental, and clinical data. 53% (8/15) of the articles identified criminally exploitable biotechnologies associated strongly with synthetic biology; split between those concerning the modification of organisms (33% 5/15) and those examining genetic modification (20% 3/15), such as gene drives and the low-cost, easy-to-use bacterial clustered regular interspaced short palindromic repeats (CRISPR) genome editing technology (Ran et al., 2013). Finally, 27% (4/15) of articles related to biotechnology systems integrated within the cyber domain; one of which assessed criminally exploitable risks associated with digital microfluidic biochips (DMFB). DMFB is hardware built to manipulate micro-droplets for biological analysis, otherwise known as lab-on-a-chip.

### Thirteen Exploits Identified

We next sought to identify what exploits were described in the 15 screened articles. For this, we conducted a content analysis of the data extracted from the 15 screened articles through the SR process (see Supplementary Table 1 for extracted data). Three concepts emerged from the content analysis: exploits, domain, and crime types. Exploits refer to the method used to commit an offense. Domain refers to the general category of crime (see below). Crime types describe the specific emerging crime opportunity facilitated by the introduction of a new technology as identified in this review.

A total of 13 exploits were identified, collated in Table 2. Two articles (Wintle et al., 2017; Kirkpatrick et al., 2018), mentioned over-lapping exploits. To highlight the most feasible exploits, we sorted the screened articles by study design (Materials and Methods). Studies that employed an experimental design were placed at the top and those that employed a speculative design at the bottom.

#### Three Domains Emerge

**Figure 4:**
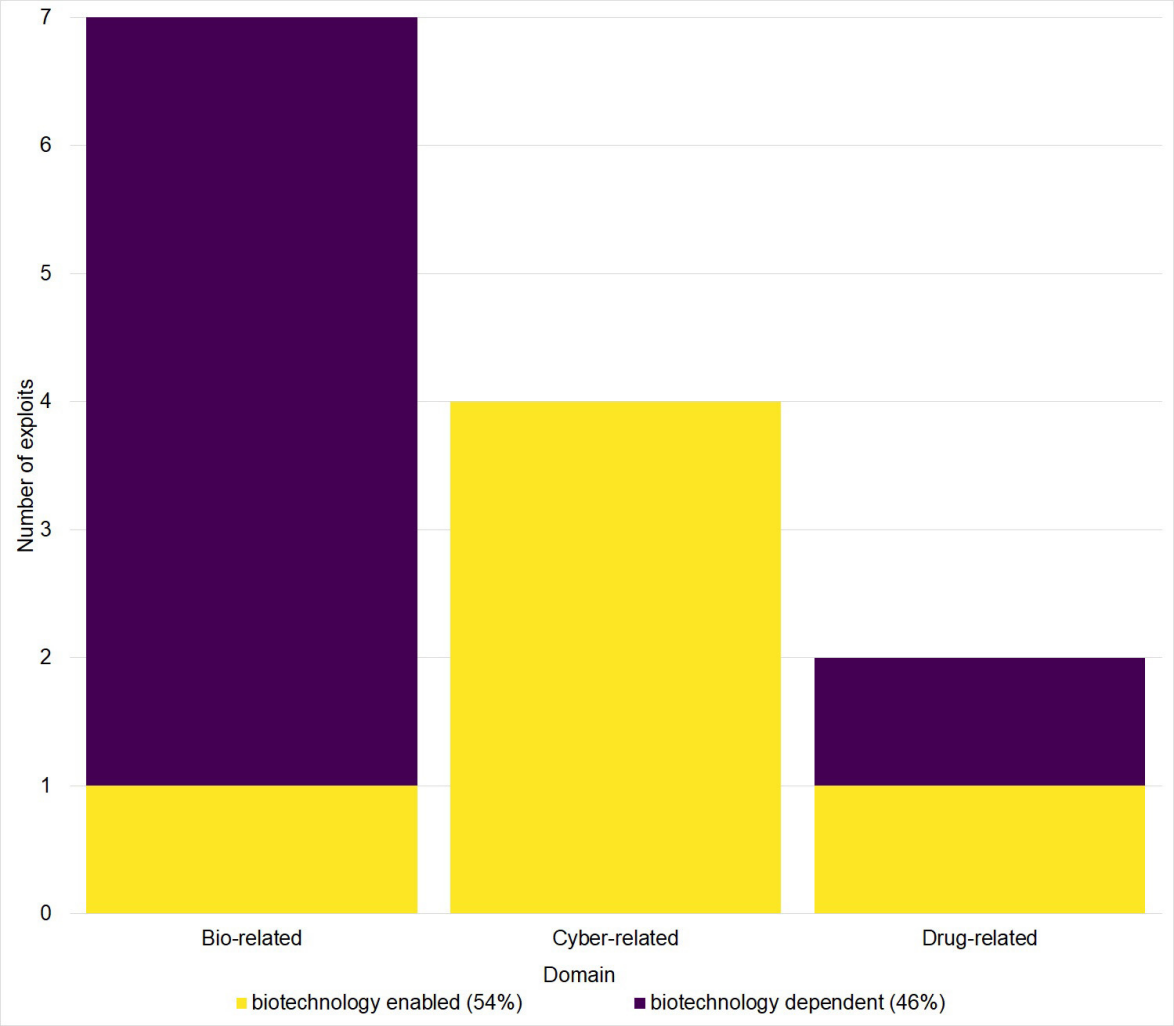
Emergent synthetic biology crime domains. Distribution of the 13 exploits identified categorized as: Biocrime, cybercrime, or drug-related crimes. Bars are color-coded to differentiate crimes that cannot be committed without the use of biotechnology (Biotechnology-Dependent crime) and those that represent traditional crimes that have been extended by the introduction of biotechnology (Biotechnology-Enabled crime).

We grouped the 13 exploits (Table 2, Figure 4) into three domains: bio-related, cyber-related and drug-related. Biocrime is the exploitation of biological tools, devices, information, or systems. Cyber-crime is that which is committed using information technologies (IT). Drug crime relates to the use and distribution of drugs and the relative degree of harm caused as defined by the Misuse of Drugs Act 1971. Further analysis of Figure 4 revealed two over-arching types of crime: Biotechnology-dependent; those that cannot be committed without the use of biotechnology, and Biotechnology-enabled; traditional crime types that have been extended in scope in some way by biotechnology. Fifty four percent of the crime exploits collated in Figure 4 were “Biotechnology-enabled” and 46% were “Biotechnology-dependent.”

#### Eight Crime Types Identified

**Figure 5:**
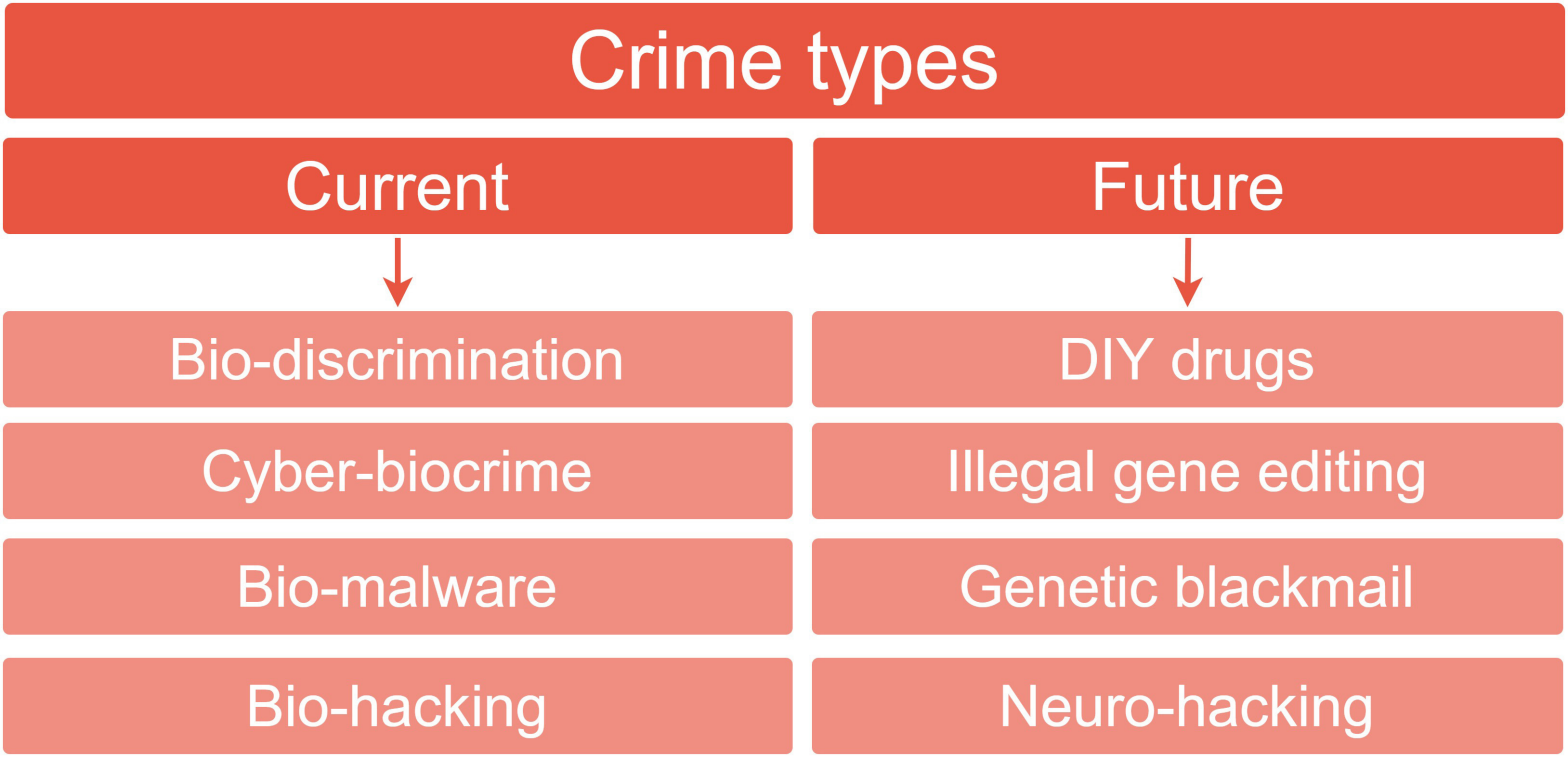
Currently occurring crime types and future predictions. The 13 exploits extracted from the 15 synthesized studies (Figure 1) were analyzed with respect to the crime types discussed as being currently feasible (left hand column) or discussed in terms of a prediction of their future emergence (right hand column).

We were also interested in the more general emerging crime types and grouped the 13 exploits according to their similarity. For example, two studies referred to privacy concerns regarding biological data: in one case human microbiome data (Franzosa et al., 2015), the other human epigenetics data (Backes et al., 2016) (Table 2). If we define a crime type as equating to such themes, a total of 8 crime types emerged (Table 2, Figure 5). These were further divided into current and future threats and are discussed in more detail below.

##### Current crime risks

1. Bio-discrimination

Databases of health/biological information are used to inform synthetic biology applications such as medical diagnostics (Slomovic et al., 2015). Such data contains sensitive information about an individual (e.g., genetics revealing one's predisposition to certain severe diseases, kinship, and ethnicity), which can lead to various forms of discrimination. “Bio-discrimination” is the use of biological data for the discrimination against different categories of people, on the grounds of their biological information. Five studies discussed the privacy concerns that emerge from the use of biological data that is now found in open source databases and also held internally in healthcare systems. These sources of data can currently be exploited to leak information on patient/individual records, enabling Bio-discrimination.

Ayday et al. demonstrate how a patient's genetic data, is exposed to an attacker at the medical unit or directly at the data storage and processing unit when calculating patient disease-risk (e.g., coronary artery disease) and propose a framework in which patients' genomic data is securely stored and processed (Ayday et al., 2013). Qu also discusses privacy concerns of genomic data as it can enable inferences regarding adverse reactions to common drugs or ancestry. Moreover, genetic data is commonly shared between repositories, is often publicly available and can be managed by third parties remotely, which can provide an effective vector for spreading malicious files. For example, Ney et al. (2017) found that 13 commonly used DNA analysis tools, that users can employ to upload and process data, are susceptible to buffer overflow attack opportunities (overwriting the memory of an application) which can be used to expose private information.

Backes et al. were able to identify and match anonymized patient records using epigenetic profiles found online. Epigenetics is the study of changes in genetic expression induced by environmental factors (such as diet and pollution) without changes in the genetic code (DNA base-pairs) (Goldberg et al., 2007). The authors assume an adversary model in which unauthorized access to epigenetics profiles of individuals is gained from a private health insurance or hospital database. This data is then matched with a public research dataset of epigenetic profiles at different time points revealing associations between non-anonymized and healthy profiles and anonymized profiles known to be associated with diseases. The result can enable attacks targeted toward high-profile victims where their health/disease data are sold and extorted for ransom. One of the attack models tested by the authors produced a 90% success rate in matching profiles. On a more positive note, the authors also propose countermeasures and show these to decrease the ability of linking the profiles by at least 50% (Table 2, Supplementary Table 1).

Harnessing the microbiome in diagnostics and therapeutics is a major research theme in synthetic biology (Dou and Bennett, 2018). Franzosa et al. were able to de-anonymize microbiome study participants, whose data is available in the public domain. The microbiome is the community of organisms (e.g., bacteria, viruses) that reside inside and on the human body that help carry out processes that humans did not need to evolve on their own, such as digestion from the gut microbiome (Turnbaugh et al., 2007). The gut microbiome is currently studied by analyzing collected fecal samples, of which data resides in public databases such as the Human Microbiome Project (https://hmpdacc.org/). Despite dynamic changes in the composition of the gut microbiome (subject to dietary intake for example), Franzosa et al. were able to uniquely identify >80% of individuals up to 1 year later using algorithms (Table 2, Supplementary Table 1), enabling bio-discrimination.

2. Cyber-Biocrime

Further to databases, synthetic biology involves a biomanufacturing process where a desired physical effect, such as synthetic drug production, is induced. This increasing integration and dependence on the digital space (for example, computer-controlled instruments within biomanufacturing processes) creates a new category of risks between cyber and biological systems. Cyber-biocrime describes criminal activities carried out by combined means of computers/Internet and biological/biochemical material, and was discussed in six studies (Ney et al., 2017, 2018; Wintle et al., 2017; Peccoud et al., 2018; Faezi et al., 2019; Qu, 2019). Peccoud et al. introduce the need for “cyberbiosecurity” to prevent (for example) the manufacture of nefarious products through the tampering of electronic orders of DNA sequences or the interception of shipments. Wintle et al. discuss robotic “cloud labs” that translate digital “instructions” into biological systems without human oversight. Absent adequate security, such advances in automation could create opportunities for crime such as the sabotage of vaccine or drug production. Additionally, Qu discusses the security of genomic data stored using cloud computing or other biobanks that are vulnerable to attacks during data transfer (for example) (Supplementary Table 1).

Ney et al. (2018) demonstrated targeted “mis-genotyping,” where a healthy DNA sample may be misclassified as one (in their example) with anemia (Figure 5, Table 2, Supplementary Table 1). This was achieved by a physical side channel attack on modern next-generation DNA sequencers (NGS) that are capable of sequencing multiple sequences at once to increase throughput. Unlike other exploits, side channel attacks work when the computer system functions normally (rather than changing its actions), but its functioning allows “eavesdropping” and the harvesting of information that is supposed to be kept secret. To allow the parallel sequencing of DNA, NGS uses unique indexes to identify the fragments of DNA as they are processed. The authors demonstrated how this process can be exploited by incorrectly assigning samples to the wrong indices, a phenomenon called “index-crosstalk.” When this occurs, information is leaked between samples and misclassifications can be generated.

Faezi et al. show that an exploit of the sound produced by DNA synthesizer machines during sequence synthesis can enable the theft of propriety (and potentially dangerous) data (e.g., sequence of a highly contagious virus). The authors demonstrate that such confidentiality breaches can be conducted with over 80% accuracy, taking only 56 h and $300 to achieve, an attack model that introduces a plausible business model for malicious actors.

3. Bio-malware

Synthetic biology workflows comprise the synthesis of complex systems with functions non-existent in nature or that modify natural systems for useful purposes. The threat of Bio-malware (*bio*logical *mal*icious soft*ware*) in the form of “trojans” was demonstrated and discussed in three papers (Ali et al., 2016; Ney et al., 2017; Wintle et al., 2017). “Trojans” are a form of malware that are used to obtain unauthorized access to or otherwise compromise systems (Kramer and Bradfield, 2010). Wintle et al. discuss the extension of well-recognized information security threats into new digital DNA tools and services that emerge (that can themselves be hacked and tampered with).

Ney et al. (2017) demonstrated that it was possible to compromise a target software system using malware stored in physical DNA. This was achieved by encoding a known exploit into the four nucleotides of DNA (A, C, T, G) to make “DNA-encoded malware.” The authors also artificially introduced a vulnerability into the DNA analysis software such that it would be triggered by the malformed DNA. The sample was then synthesized in a typical manner using Illumina Sequencing to generate the reconstructed sequences in digital form (FASTQ files). Once read, the files were executed, and the designed DNA exploit enabled remote access to the system. While this was an orchestrated attack in that the authors introduced the vulnerability for the deployed DNA-encoded malware, this approach, of anticipating or simulating scenarios of adversarial behavior, remains rare in both bioinformatics and synthetic biology.

Unlike Ney et al. who demonstrated the threat model experimentally, Ali et al. speculated on the vulnerabilities associated with the security of digital microfluidic biochips (DMFB). Through the analysis of the cyberinfrastructure supply chains used in the production of DMFBs they identified targets for Trojan attacks. Successful implementation of these attacks by an adversary could enable the manipulation of assay outcomes (e.g., results from point-of-care medical diagnostics, DNA sequencing, and airborne particulate-matter detection), leak sensitive information or damage the microfluidic device making it unusable.

4. Biohacking

Synthetic biology has been democratized in major part due to the rapid decline in the cost of custom DNA synthesis (Serrano, 2007). Four studies (Dieuliis and Giordano, 2017; Wintle et al., 2017; Kirkpatrick et al., 2018; Bress) discussed how this enabled biohacking and tailored drug manufacturing (Figures 3, 5). Bress defines biohacking as “*the process of exploiting or tinkering with genetic material of existing organisms*” including “hacking” into the brain through the abuse of nootropics (cognitive enhancing drugs) to improve performance. Historically, athletes have abused steroids and growth hormones (Reardon and Creado, 2014). Similarly, cognitive enhancing drugs (nootropics) are currently being over-prescribed, with (for example) a reported abuse rate of 43% in Attention Deficit Hyperactivity Disorder medications which enrich attention, motivation, and focus while decreasing fatigue (Advokat et al., 2008). Bress reports a current commercial uptake of “biohacking,” identifying companies such as Nutrahacker and Promethease that provide nootropics tailored to the customer's genetic makeup (obtained through saliva samples) for cognitive enhancement. The authors argue that the trend of biohacking will continue and present challenges to drug regulation.

Wintle et al. describe biohackers as new “makers” that are expecting to disrupt pharmaceutical markets by (for example) developing open source and inexpensive generic insulin from bacteria. Kirkpatrick et al. discuss the activities of biohackers involving (for example) them publicly injecting themselves with genetically engineered compounds (e.g., N6 gene to produce HIV antibodies, myostatin gene for muscle growth) that they have experimentally produced on their own using CRISPR/Cas technology—in unregulated premises. Kirkpatrick et al. also discuss biohackers' enterprises, namely the Odin and the Transcendence research collective. The former was founded to sell CRISPR/Cas kits for “at-home” experiments containing unregulated CRISPR/Cas constructs. The latter was founded to conduct “open clinical trials” or a means by which biohackers can test the gene therapies that they have developed; through “self-experimentation.” Although self-experimentation might pose serious public health concerns, Kirkpatrick et al. argue that the lack of oversight in these markets provides more opportunities for acquisition of information and materials, such as gene therapy delivery vectors, that could be rate limiting steps in malicious applications. DiEuliis et al. discuss the international Genetically Engineered Machines (iGEM) competition as a concern. The sophisticated projects presented at iGEM in their example could bring future risks of an amateur accidentally or purposefully creating a harmful entity (e.g., virus) using CRISPR/Cas techniques.

##### Future crime risks

5. DIY-drugs

Synthetic biology engineering involves modifying a biological system, such as bacteria, to produce a desired active ingredient that may not be produced naturally; introducing “Do-It-Yourself” (DIY) drugs. Motivated by the current commercial uptake of “biohacking as-a-service,” drug manufacturing “at-home” using genetically modified organisms was identified as a future crime risk in two studies (Wintle et al., 2017; Bress). Through scenario development, Bress forecasted that by 2030, synthetic biology will facilitate illicit drug abuse. The scenario developed was based on research that used genetic engineering tools to modify *E. coli* and yeast to produce Tetrahydrocannabinol (THC) and Lysergic acid diethylamide (LSD), respectively (Endy et al., 2015; Luo et al., 2019). Bress predicted that it will be commonplace in the future for synthetic drugs to be commercially cultivated or made “at-home.” She speculated that this will be fueled by an increasing use of nootropics. Moreover, that crime opportunities will be generated by asymmetries in policies across countries (i.e., use/production will be legal in some countries but not others) that will create opportunities for drug trafficking. An analogy can be made here to the current (asymmetric) legislation regarding cannabis use in the U.S. which differs across States. Bress suggests that the production of illicit drugs using synthetic biology techniques (without having to cultivate fields of plants) would disrupt drug trafficking and economic incentives for drug manufacturers. It would, she suggests, enable the production of desired substances in a petri-dish, equipping any individual with the capability of using, producing and distributing manufactured drugs, potentially decentralizing drug trafficking.

The economic benefits of using fast-growing, genetically modified microbes as drug manufacturing platforms have been a feature of conventional drug manufacture for decades. In their horizon scanning and Delphi study, Wintle et al. suggest that the manufacturing of illegal drugs using fast-growing genetically modified microorganisms (e.g., yeasts or bacteria), represent an increasingly attractive model for criminals as the barrier to entry steadily decreases. They predicted this will facilitate the unlicensed production of illegal substances at lower costs but with potentially less purity in comparison to pharmaceutical products. Like Bress, Wintle et al. predicted that this will either change existing drug transit routes and crime networks or be adopted by existing criminal networks (Oye et al., 2015).

6. Illegal Gene Editing

Genome editing tools (König et al., 2013), such as CRISPR (Rowe et al., 2020) and transcription activator-like effector nuclease (TALENS) are strongly characteristic of synthetic biology (Moore et al., 2014). These tools can be used to edit the genomes of organisms extensively without inserting additional genetic material (Waltz, 2016), or as assembly tools to compose and insert complex synthetic gene networks into cells (Kim et al., 2019). Two studies discussed gene editing with respect to legislation regarding future applications, such as human enhancement in the form of cosmetic changes or military capabilities for “super soldiers.” Wintle et al. and Fears et al. raised concerns about the risks to future generations who cannot consent to such changes, as the decision is made for them since genetic edits are heritable. Wintle et al. also discussed socioeconomic concerns associated with the future cost of genetic enhancement, noting that it may not be available for all societal classes or affordable for governments to provide though public healthcare. This could introduce the emergence of black markets offering such services absent regulatory oversight. Kirkpatrick et al. discuss reckless applications of CRISPR/Cas in cosmetics to produce a market of unapproved medical treatments that follows a similar pattern to that of stem cell clinics in the U.S.; where 570 clinics in 2016 were found to offer unapproved treatments for medical conditions and for cosmetic enhancement.

Three studies (Hauptman and Sharan, 2013; Fears and ter Meulen, 2018; Kirkpatrick et al., 2018) identified illegal gene editing in the context of dangerous pathogens (e.g., viruses). Hauptman et al. refer to the increasingly accessible genetic engineering tools that can be used to make more virulent pathogens. Kirkpatrick et al. speculate dual-use concerns on the State who in their example would use published research from academia to pursue covert assassinations, such that slow and natural-looking death is induced but goes undetected as a crime. Additionally, in a scenario-building exercise, the security implications of privately funded and rapidly increasing biotech startups were discussed. In one discussion Kirkpatrick et al. propose a route whereby indirect, or rare, mechanisms for favoring the generation of effective human pathogens could also be exploited to cause harm. As an example of such a rare and non-obvious mechanism, they discuss how partially humanizing livestock, to engender resistance to native viruses, could favor the emergence of human-specific pathogens. The rarity of these occurrences or mechanisms for human pathogen production could, in such cases, provide cover for those intending harm.

7. “Neuro-hacking”

The interaction between the host and gut microbiome can influence the central nervous system, modulating the brain and behavior, including mood (Muller et al., 2020). Synthetic biology is increasingly seeking to modulate, control and even design (Alnahhas et al., 2020) the microbial consortia that form microbiomes. Synthetic organisms can be used as medicines, engineered to perform metabolic functions that once ingested could treat disease (West et al., 2018). Physiological processes are connected such that alterations to biological activity are relayed to the brain to induce behavioral changes, when necessary. For example, gastrointestinal activity can induce stress, sensations of nausea, pain and satiety (Forsythe and Kunze, 2013). This is due to the peripheral connection of the gut to the brain through the enteric nervous system but also through the specific responses induced by the composition of the gut microbiome and the species (e.g., bacteria) it contains.

The interactions between the gut microbiome and the brain can theoretically be exploited as described in three studies. DiEuliis et al. defined it as “neuro-hacking.” These studies predicted that neuro-hacking could be performed by the manipulation of bidirectional signaling between the gut microbiome and the nervous system or through the development of targeted microbiological/pharmacological activators/effectors of neural function. Neuro-hacking would therefore be used to induce changes in behavior rather than to cause fatalities (unlike neuroweapons). The use of genome editing tools to target microbiome components to cause purposeful imbalances were discussed by Kirkpatrick et al. in a scenario-building exercise. In their example, these imbalances would cause a perturbation of a healthy microbiome to provide a “backdoor” to other physiological systems that are linked, such as the immune system. For example, Kirkpatrick et al. discuss the scenario of the covert use of a viral vector that crosses the blood-brain barrier to target the brain (memory and cognitive functions) and eventually causes death without the perpetrator being detected. Wintle et al. forecasted developments in therapies for microbiome manipulations. A form of this already exists commercially in probiotics and prebiotics intended to induce a “healthy” balance of the gut microbiome. In the future and with more advanced microbiome-therapies, neuro-hacking may be exploited for malicious purposes.

8. Genetic Blackmail

Health/biological information in digital databases that are used to inform synthetic biology applications such as medical diagnostics and drug discovery, travels through integrated workflows. Such databases now also include millions of consumers' genetic information from commercialized DNA testing kits (e.g., Ancestry DNA and 23AndMe). Data breaches and vulnerabilities in internet protocols—that make bioinformatics tools, shared databases, and cloud computing of genomic data insecure—were identified in four studies (Hauptman and Sharan, 2013; Backes et al., 2016; Wintle et al., 2017; Qu, 2019) (Table 2, Supplementary Table 1, Figure 5). Qu's literature review raised issues around “Genetic blackmail” or the act of coercion using the threat of revealing an individual's genetic information unless certain demands are met. Qu discussed how genetic blackmail can be achieved through (for example) the combination of publicly available (anonymized) genetic data from the genetic-genealogy database “Ysearch” and a public record search engine such as PeopleFinders.com or, by exploiting network vulnerabilities as these databases do not use a secure channel such as Secure Socket Layer (SSL). SSL is a protocol that protects sensitive information (e.g., passwords, credit card details, health data) from hackers through encryption which ensures that only the intended recipient can access it when information is sent over the internet (Wagner and Schneier, 1996). Qu discussed the fact that using “shodan.io” (a search engine that indexes connected computers, including servers), adversaries can view data exchanged between universities and research institutes and uncover sensitive data by “sniffing” packets.

Moreover, vulnerabilities exist in the databases themselves (Gu et al., 2019). For example, there are authentication weaknesses in databases (such as the large and cross-platform MongoDB database program) that can be exploited through cyber-attacks (described in more detail in Table 2, Supplementary Table 1). This concern is particularly pertinent in the U.K., for example, as information from the U.K. government-owned company Genomics England currently resides on MongoDB. Qu also highlights the National Cancer Institute (NCI) Cancer Genomics database, which is hosted on the cloud. The use of cloud services introduces problems when the data is not encrypted in storage or transmission. Security problems already identified for internet connected or Internet of Things (IoT) devices such as data breaches (Andrea et al., 2015) are expected to impact biotechnologies (Hsu and Sandford, 2007; Thomas and Harden, 2008; Smith et al., 2011; Lewis, 2019). For instance, concerns about information security were raised regarding the digitization of DNA, the hackability of DNA sequencing and the decentralization of bio-automation (Wintle et al., 2017). More generally, the integration of cyber and physical processes in biotechnology workflows were identified as introducing vulnerabilities in security. This integration makes common cyber-attacks, such as network attacks possible, but creates opportunities for attacks on biomanufacturing processes (bioreactors, fermenters, and other biological, chemical, and physical processes) as these are increasingly controlled by computers (Peccoud et al., 2018).

The lack of basic cyber hygiene (Table 2, Supplementary Table 2) and ease of access to systems generate the propensity for “The Genetic Blackmailers,” identified in a scenario-building exercise (Hauptman and Sharan, 2013) by Hauptman et al. Described as a “wild-card” scenario (low-probability, high-impact), an individual would misuse DNA information for extortion. Examples the author's listed included incorrect criminal profiling based on planted DNA and paternity suits against a billionaire using fabricated (synthetic) DNA samples. Insurance company exploits, which would involve calculating insurance rates according to fraudulent genetic samples, “DNA-phishing” (i.e., identity theft from discarded physical DNA samples) and the “removal of inferior life” and/or “optimization” of human beings using genetic technologies, were also mentioned. Further details of these scenarios could not be found online or from contacting the authors.

### Five Crime Influencing Factors

**Figure 6:**
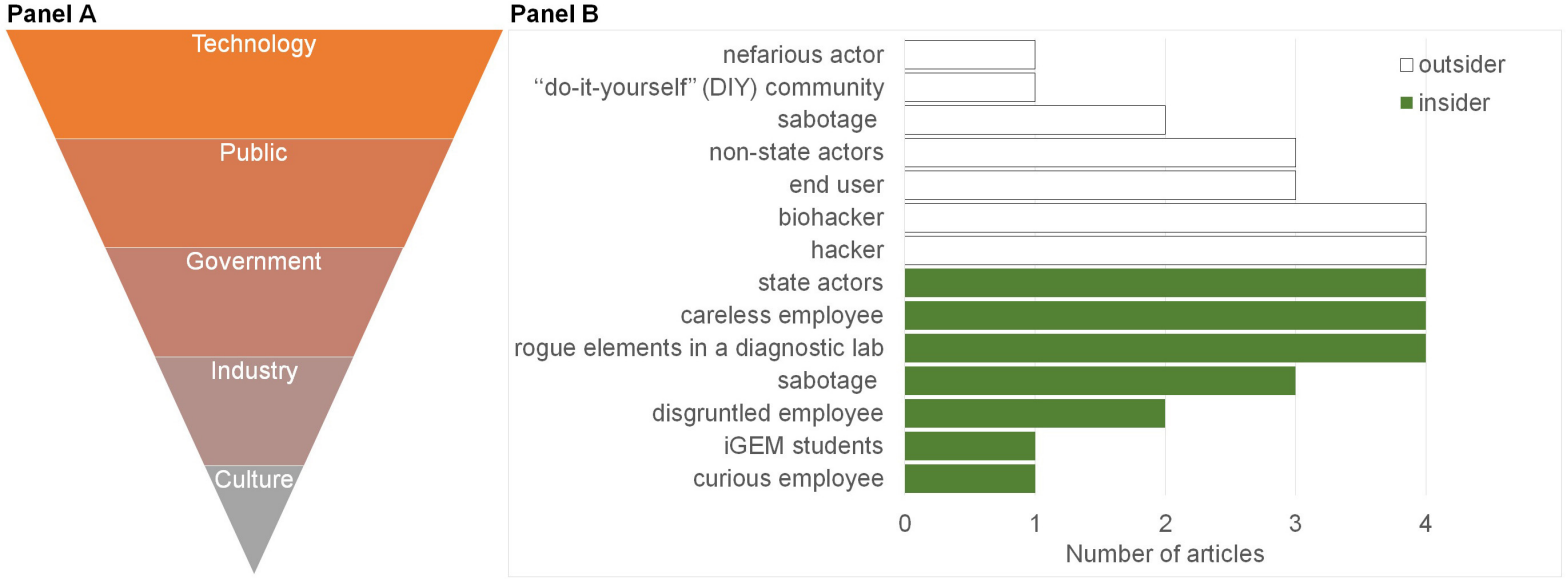
Influencing factors and offender threat models. The 15 reviewed studies (Figure 1) were analyzed with respect to the Influencing Factors **(A)** for crimes enabled by synthetic biology and Offender Threat Models **(B)** they described. **(A)** Five Influencing Factors were identified and are ranked here top to bottom in order of their greater to lesser prevalence (Supplementary Table 2) in the 15 screened studies. **(B)** A bar graph of the distribution of articles by the offender threat model discussed. “Insider” (green) threat models were defined as someone who has access to information that would otherwise require authorization. Outsider threat models (white) were defined as someone who would need to gain access from the outside.

The conditions that may contribute to making the crimes described possible were extracted and sorted according to the frequency with which they were discussed (Figure 6A, Supplementary Table 2). Five main influencing factors were identified. These were changes in the activity of government, technology, industry, culture, and public perception that could influence the crime event. Most changes identified concerned technological factors including hyper-connectivity and increases in the number of devices (such as IoT), as well as cloud computing and increased automation (Hauptman and Sharan, 2013; Ney et al., 2017; Qu, 2019; Bress). The discussion of public perception considered the need for biotechnology literacy, the necessity for more awareness of risk and the general activity of the user. Regulation and accelerated funds within defense research were some of the issues discussed in relation to government. Apropos industry, discussion included the increasing numbers of commercial service providers (Hsu and Sandford, 2007; Turnbaugh et al., 2007; Ali et al., 2016; Murch et al., 2018; Ney et al., 2018; Lewis, 2019) who prioritize profit, possibly at the expense of security. This is exemplified in the case of the IoT (DDCMS, 2018; Blythe and Johnson, 2019) and is expected to extend to biotech start-ups. Finally, cultural shifts toward open source biology were also discussed (Franzosa et al., 2015; Backes et al., 2016; Wintle et al., 2017; Fears and ter Meulen, 2018) along with the challenges this may present to policy-makers. Open source biology and “generative” biotechnology (e.g., CRISPR/Cas) is advantageous as it accelerates experimentation and innovation—both in terms of the wider range of applications to which it is applied, and the diversity of users who use it. At the same time, open source biology increases the attack surface of crime, especially as biotechnology is increasingly integrated into the global economy and introduces new attack vectors that surpass the current biosecurity paradigm of shortlisted pathogens.

#### Fourteen Offender Threat Models

Some commonalities emerged in the threat models described and demonstrated in the articles. These were divided into “insider” (Figure 6B, green) and “outsider” threats (Figure 6B, white) to distinguish the source of security vulnerabilities in an attack surface. An insider is someone with access to information that would otherwise require authorization, whilst an outsider is someone who would need to gain access from the outside (Pfleeger, 2008). Of the five main offender threat models (>3 mentions), 40% of the articles identified hackers and biohackers as the most probable outsider threat, whilst insider threats were identified as rogue elements in a laboratory, or careless employees (who might enable crime, rather than commit it) and State actors.

## Discussion

The scale of security vulnerabilities associated with synthetic biology remains to be quantified. However, developments in synthetic biology, like any other technology, occur with such speed that security is often overlooked. This SR revealed evidence on current and predicted crime themes found in the literature, highlighting research gaps, the need for multidisciplinary crosstalk, the currently exploitable biotechnologies, the emerging (and future) crime opportunities and points for intervention.

The Research Gap is evident (Figure 1—low number of articles extracted). The resulting papers that comprised the synthesis of this review were all recent (2013–2019), indicating that concerns about security in this field are only now surfacing (Figure 2). Most publications were from the U.S., which could be a result of heightened concern from the U.S. Intelligence Community, the director of which in 2016 had just labeled genome editing as a national security threat (US Department of Homeland Security, 2016). However, studies must extend beyond the U.S., as synthetic biology threats do not conform to borders, and practices and opportunities may vary across them.

There is a clear need for more multidisciplinary crosstalk. Only two of 15 papers were a result of collaborative work (Figure 2), but the threats identified were not limited to disciplinary boundaries. Less than half of the studies were “experimental” (i.e., had quantitative study designs presenting a proof-of-concept) and none were “currently occurring in the wild” (i.e., study design demonstrating the crime in the real world). We see the latter as a positive. However, the absence of reports does not mean an absence of the problem. Indeed, crime facilitated by synthetic biology may currently be under-reported as its forms may be unknown or undetectable.

For the vulnerabilities that are known, there is an urgent need to improve basic cyber hygiene by educating researchers within the life sciences and to provide them with the technical skill set and tools necessary (e.g., more secure storage and handling of data as well as authentication controls when necessary) (Figure 6A, Supplementary Table 2). There are no current frameworks in place to address the cyber-bio-infrastructure and the outcome of this review highlights the need for one in the context of crime prevention. Absent one we risk a crime harvest occurring in the future.

Emerging crime types found in this review were mainly bio-related, cyber-related, and drug-related (Figure 4). Currently feasible crimes were found to be in the form of bio-discrimination, cyber-biocrime, bio-malware and biohacking (Figure 5). All relied on cyber-controlled processes and all were shown to be preventable through increased cyber-hygiene. Future crimes posed a challenge in identifying relevant prevention techniques. DIY-drugs, illegal gene editing, genetic blackmail, and “neuro-hacking,” heavily depend on our currently limited understanding of complex biological systems, public perception and regulation.

**Figure 7:**
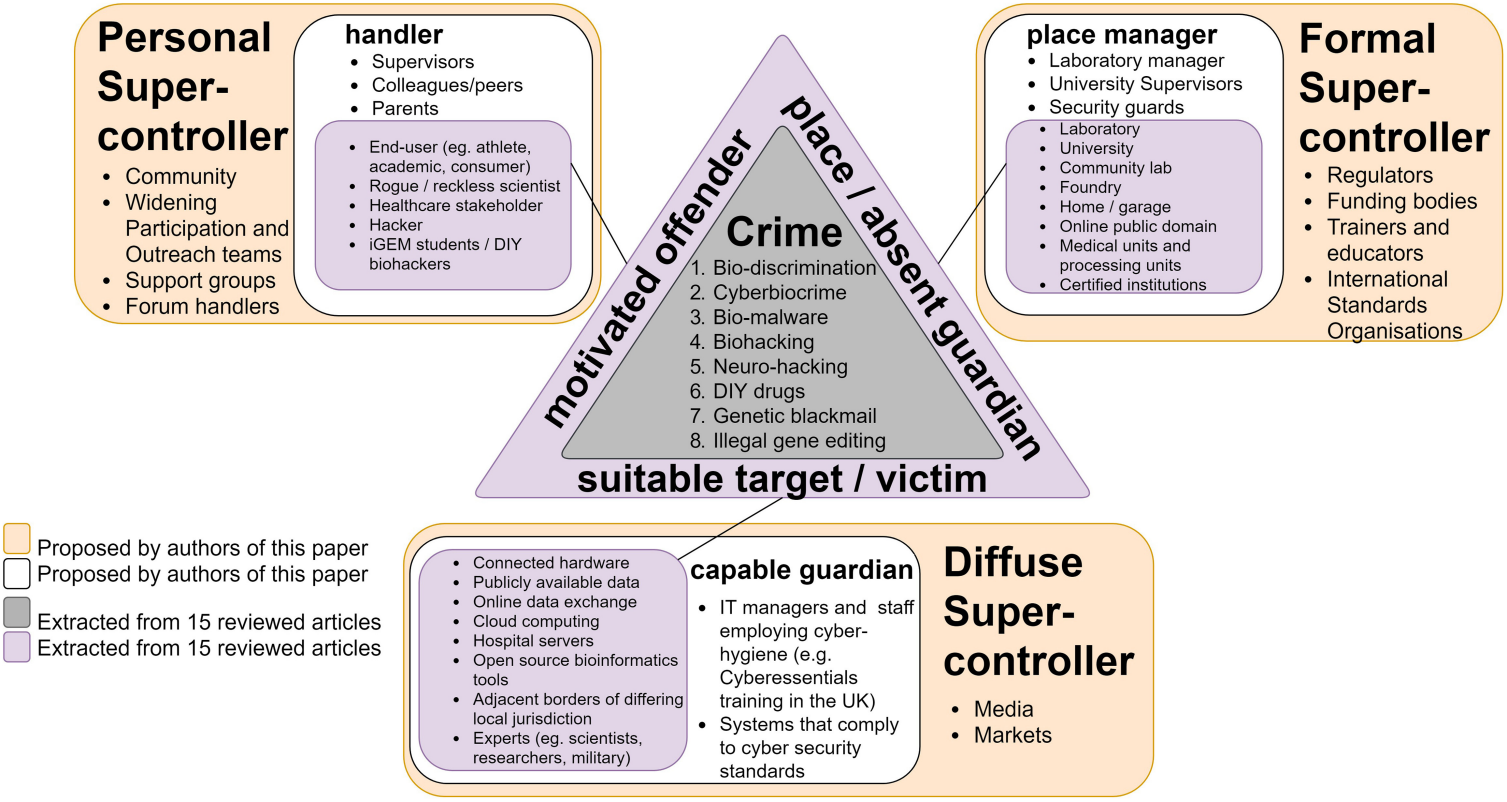
Eck's triangle of synthetic biology crime opportunity landscape. Eck's triangle (Eck, 2018) is used to summarize the elements required for synthetic biology crime to occur as described in the 15 studies (Figure 1). For the synthetic biology-related crimes to occur (gray area at the center of the triangle), three elements must converge (purple): a motivated offender (left-hand side of triangle) and suitable target (bottom of triangle) need to converge in space and time in an unguarded place (right hand side of triangle). Absent this convergence, crime is unlikely or even impossible. Each element has a “controller” (white box) that can influence these interactions. Referred to as handlers, guardians, and managers, these are people (or things) that can influence the actions of offenders, protect targets and manage places in some way, respectively. These controllers are in turn influenced by “super-controllers” (orange boxes). Super-controllers can be divided into three general categories of actors: those that have formal, diffuse, and personal influence, and they can include governments, the media and the family, respectively. The crime types, motivated offenders, suitable target/victim, and place/absent guardian were extracted from the 15 reviewed articles. The controllers and super-controllers are proposed by the current authors as examples and are not exhaustive.

Through a Crime Science lens and using the routines activity approach (Felson and Cohen, 1980) (a framework for thinking about what and who might influence the likelihood of a crime event) the crime types identified were considered in terms of the ecosystem in which they might occur to identify potential points for intervention (Figure 7). Five main factors were identified (Figure 6A) that if targeted could offer opportunities for disrupting the current and forecasted crimes. Figure 7 shows how we applied the routine activity approach in the context of the crime types identified in this review. As the number of “super-controllers” within synthetic biology is likely to be accounted for by a small amount of people and places (Farrell et al., 1995; Sampson et al., 2010), there are only a few actors that can at this point control/contribute to the security and use of the technology. As an example, 80% of worldwide gene synthesis capabilities occurs by company members of the International Gene Synthesis Consortium (IGSC), such as Twist Bioscience, ThermoFisher Scientific and GenScript (Kobokovich et al., 2019). Therefore, targeting super-controllers can play a role in the effective crime prevention of common future crime forms within synthetic biology. We suggest the following:

- **Formal Super-controllers** (regulatory, financial, and organizational) can address (current) loose and fragmented global regulation (Kirkpatrick et al., 2018) for securing cyber-physical interfaces (Wintle et al., 2017; Peccoud et al., 2018; Faezi et al., 2019) in biomanufacturing process and workflows in the pursuit of a bioeconomy (Wintle et al., 2017). This can be achieved by reviewing, implementing, and enforcing clear regulations, accelerating the funding in experimental research (“ethical hacking”) and innovation with the goal of identifying and addressing risks; financing community labs and activities that engage in responsible research and innovation and increasing training and education *between* disciplines (e.g., Life sciences, Information Technology, and Ethics) (Ali et al., 2016; Fears and ter Meulen, 2018) for diversified but responsible research.
- **Diffuse Super-controllers** (markets and media) In a hyper-connected/hyper-personalized market with an increase in providers and public access, higher security standards, and quality assurance frameworks may better protect the public from emerging technologies and their implications (Ali et al., 2016; Fears and ter Meulen, 2018; Bress); engaging with the media to increase awareness of the risks (and expected standards) to the public, but also implementing channels for the safe reporting and recording of events (vulnerability disclosure).
- **Personal Super-controllers** (groups) To encourage links and activity in community labs, to engage with a diversity of groups (e.g., biohackers and hackers) to support an open source culture and experimental research (Franzosa et al., 2015; Backes et al., 2016; Wintle et al., 2017; Fears and ter Meulen, 2018), while enhancing communication channels of findings (both positive and negative) and responsible research and innovation.

## Limitations and Future Research

Articles were limited to those written in the English language, and as such articles from countries known to have activity in this space (e.g., China, The Netherlands) may not have been represented. This may have created a language bias (Egger et al., 1997; Egger and Smith, 1998; Morrison et al., 2012; Cockbain et al., 2018), and may also contribute to the low number of articles found in the academic literature. We aim to address this and the research gap by conducting a parallel study. This will take the form of Delphi study with field experts (with an international network), who will be asked a set of questions across multiple rounds, with the aim of forecasting crime trends.

## Conclusion

Currently, biosecurity is outdated and until dedicated resources are allocated to address these crime types as something distinct, it will likely be outpaced. Despite the concerns raised about the misuse of synthetic biology, no previous work has been conducted from a Crime Science perspective to systematically collate and assess the academic literature. A three-step screening protocol was previously devised (Elgabry et al., 2020) and here applied to extract all peer-reviewed academic literature on exploitable biotechnologies. An initial search query yielded 794 entries, but only 15 fully met the inclusion criteria. All 15 articles were published between 2013 and 2019. This signals the embryonic stage of the field of biosecurity and an urgent need for more work. That most studies were published by researchers in the U.S. also signals the need for more international work on this topic.

The most common crime opportunities emerged through insecure biological data, synthetic biology technologies and manufacturing workflows (Figure 3). Synthetic biology technologies were associated with 46% of the crime types identified (Figure 4). Forty percentage of the most common outsider threats for synthetic biology crime identified were biohackers and hackers (Figure 6B). Three articles identified by our SR protocol (Hauptman and Sharan, 2013; Fears and ter Meulen, 2018; Kirkpatrick et al., 2018), detailed malign use of engineered viruses as a future crime risk. Changes in technology, government, industrial practices, and cultural attitudes were the most common enabling factors cited for synthetic biology-enabled crime (Figure 6A, Supplementary Table 2).

For an effective preventative approach against these emerging crime risks, immediate attention and a creative preventative approach is needed. The impact on global health and the world economy of the current SARS-CoV-2 pandemic has brought into sharp focus this unmet need. As a result of this SR and other work conducted in parallel, along with others (Evans et al., 2020), we propose the increase of “ethical hacking” (Pashel, 2007) as a way to move away from reactive changes (implemented after major events occur) to proactive governance in health security and biosecurity. We suggest this can be achieved by applying the hacker ethic of Information Technology in the Life sciences, to iteratively test the boundaries and experiment on new synthetic biology technologies, generating a dynamic understanding of their security limitations. To compliment ethical hacking, a “vulnerability disclosure policy for laboratories” should be enforced, similar to that proposed by the Department for Digital, Culture, Media and Sport (DCMS) in the U.K. for the Internet of Things (DDCMS, 2018). This will contribute to the early detection and prevention of crime threats enabled by synthetic biology, ideally before we witness a potential crime harvest of the kind discussed here.

## Author Contributions

ME wrote the initial manuscript. SDJ and DN contributed to revising this manuscript. All authors read and approved the final manuscript.

## Conflict of Interest

The authors declare that the research was conducted in the absence of any commercial or financial relationships that could be construed as a potential conflict of interest.
