# Supplementary material for: A Systematic Review of the Criminogenic Potential of Synthetic Biology and Routes to Future Crime Prevention
Source: Front Bioeng Biotechnol. 2020 Oct 6;8:571672. doi: 10.3389/fbioe.2020.571672 (PMC7573185; doi:10.3389/fbioe.2020.571672)
Supplement: Supplementary file 1 [file Table_1.docx]

Supplementary Material

**Supplementary Table 1. Summary of findings from the studies included in the review.** Study identifiers (authors, country, date published, study discipline), study design (speculative, experimental or “currently occurring in the wild”), biotechnology in use, exploit described or demonstrated, crime type, offender threat model, main findings reported in the 15 reviewed studies. Studies are labelled by study design to signify articles that are either speculative, experimental or “currently occurring in the wild”. Articles with experimental designs demonstrate feasibility of the method used to commit the exploit whilst speculative articles discussed a potential exploit without a proof of concept. Articles that discussed reports of actual misuse in the real-world are “currently occurring in the wild”.

| ***Authors, year*** | ***Country*** | ***Discipline (Source)*** | ***Study design***  *(speculative, experimental or “currently occurring in the wild”)* | ***Biotechnology*** | ***Exploit*** | **Crime type** | ***Offender threat model*** | ***Main findings reported*** |
| --- | --- | --- | --- | --- | --- | --- | --- | --- |
| *Ayday et al. (2013)* | Switzerland | Computer Science (USENIX) | Experimental - Penetration testing using cryptographic methods on genomic data) | Genetic, Clinical and environmental data for disease risk | Privacy concerns for exploitable (sensitive) biological data (storage and processing) | Bio-discrimination | • Careless employee (insider)  • Disgruntled employee (insider)  • Curious employee (insider)  • Hacker (outsider) | Assessed (real) genetic (single nucleotide polymorphisms (SNP) profiles), clinical and environmental data that may introduce privacy concerns when used by medical stakeholders to calculate patient disease-risk (coronary artery disease). Such data can include demographics, family history and diseases carried or laboratory test results (e.g. cholesterol levels). The authors demonstrated that the calculation of disease risk for patients can be compromised by either an attacker (both insider/outsider threats) at the medical unit or directly at the data storage and processing unit. By developing a privacy-preserving framework and implementing data encryption (for example), the authors propose a framework in which  patients’ genomic data is securely stored and processed when combined with clinical, and environmental risk factors (extracted from a dataset of 2078 individuals) to calculate coronary artery disease risk. |
| *Backes et al. (2016)* | Germany | Computer Science (USENIX) | Experimental  - Penetration testing using probabilistic and algorithmic methods on epigenetics data | Epigenetics data | Linkability threat and privacy risk for exploitable Epigenetic data | Bio-discrimination | • Hacker (outsider)  • Careless employee (insider) | Matched (non-anonymised and anonymised) epigenetic (miRNA expression profile) and genetic expression profiles taken a week apart with a 90% success rate, using two types of attacks, identification and matching, on three different longitudinal datasets and one cross-sectional dataset (two of which are freely available online through the Gene Expression Omnibus (GEO) database). Identification attack identified a specific epigenetics profile in a database of multiple profiles by knowing the targeted profile at another point in time and by using principal component analysis. The matching attack tracked expression levels over time and matched the target expression profile using a minimum weight assignment algorithm. This algorithm minimizes the sum of the distances between every matched pair of expression profiles (Edmonds, 1965).  Two countermeasures were proposed: an obfuscation and a probabilistic technique. The obfuscation hides a subset of disease-irrelevant epigenetic profiles while the probabilistic technique masks expression profiles by adding noise. The latter technique was found to be a better trade-off between privacy and disease-prediction accuracy (tested on 19 different diseases). |
| *Ney et al. (2017)* | USA | Computer Science (USENIX) | Experimental - Penetration testing using physical DNA on a computer program | DNA sequencing | DNA “malware” | Bio-malware | • Sabotage (insider)  • Sabotage (outsider) | Compromised a computer program (with a known vulnerability artificially induced to it) by sequencing “contaminated” DNA. This was achieved by encoding a known computer exploit into the four nucleotides of DNA (A, C, T, G) to make a DNA-encoded exploit. This was then synthesized into physical DNA. Illumina NextSeq* was used to sequence the strands that resulted in files (FASTQ) that, once read, would execute arbitrarily remote code through a reverse shell call-back. This would give the infiltrator arbitrary remote code execution causing information leakage and the ability to inject data or reveal sensitive information.  Also tested the general security hygiene of common DNA processing programs and found buffer overflow attack opportunities in 13 tools and 10 control programs.  (*“Illumina NextSeq” - DNA sequencing machine manufactured by commercial DNA sequencing company “Illumina” capable of sequencing multiple sequences at once to increase throughput.) |
| *Faezi et al. (2019)* | USA | Computer Science (USENIX) | Experimental - Penetration testing using machine learning methods on acoustic data | DNA synthesis machines | Oligonucleotide sequence theft through an acoustic side channel attack | “Cyber-biocrime” | • Sabotage (insider)  • Sabotage (outsider)  • State actors (insider)  • Disgruntled employee (insider) | Demonstrated an exploit of the sound produced by DNA synthesizer machines during sequence synthesis. This can enable the theft of propriety data (i.e. the sequence of the DNA being synthesized). Treating the DNA synthesizer as a “black box”, the authors were able to estimate synthesized DNA sequences from the acoustic patterns emitted by the machine using a supervised classification model with two phases. In the training phase, acoustic sensors (from a phone for example) were placed close to the DNA synthesizer (where the solenoids and the pressure valves are), to capture the sound to be recorded. Pre-processing and feature extraction on the recorded audio were conducted after labelling the corresponding nucleotide base (A, C, T, G) with the acoustic emission. The sequence composition (i.e. the nucleotide bases of the sequence) was then estimated given the acoustic emissions. In the attack phase, acoustic sensors were placed at the target synthesizer, and the target DNA sequence was inferred through the recording (with over 80% accuracy).  Assumptions: the target and profiling DNA synthesizers are the same machine, attack must be non-invasive, attacker has physical proximity to the DNA synthesizer |
| *Franzosaa et al. (2015)* | USA | Life Sciences (PNAS) | Experimental - Penetration testing using algorithmic methods on microbiome data | Microbiome data | Privacy threat of exploitable Microbiome data (The gut microbiome characterises an individual with more information than genetic material (i.e. DNA), and includes diet, health status, age, and geography. The analysis of a gut sample can reveal whether an individual resides in or outside the city, if he/she co-habilitates with animals (Song et al., 2013), and recent research suggests that it can be linked to anti-social behaviour (such as Attention Deficit Hyperactivity Disorder) through the bi-directional signalling of the gut-brain axis (Gato et al., 2018). Given the additional information it provides, and albeit its impalpable use in personalised medicine, it might also be used in profiling and re-identification of individuals through unique microbial “fingerprints” that distinguish them from the population (49). | Bio-discrimination | • Hacker (outsider) | De-anonymization of microbiome study participants, whose data was available in the public domain, through the development of a hitting set-based coding algorithm that identifies for each individual, a subset of features unique among every three individuals. These were referred to as metagenomic codes or sets of microbial genes of certain feature types. Gut microbiome data was used to identify individuals up to a year later with a more than 80% success rate. |
| *Ney et al. (2018)* | USA | Life Sciences (BIORXIV) | Experimental - Penetration testing using wet laboratory techniques | DNA sequencing | “Targeted mis-genotyping" | “Cyber-biocrime” | • Hacker (outsider)  • End user (outsider)  • Careless employee (insider) | Demonstrated a laboratory exploit that took advantage of high throughput DNA sequencing methods. A physical side channel attack was deployed in which a genetic sample from a healthy individual would be misclassified as one suffering from (in their example) sickle cell disease or anaemia. This was achieved by deliberately crafting a molecular DNA sequencing library that would modify the sequencing results of samples that are pooled and sequenced together. |
| *Fears et al. (2017)* | International | Life Sciences (Inter Academy Partnership) | Speculative - Expert workshop of breakout discussions with 200 experts | Genome editing / gene drive technology | Exploitation of CRISPR | Illegal gene editing and “neuro-hacking” | • End user (outsider)  • State actors (insider) | Assessed genome editing and its implications through a global effort led by the international academy partnership (IAP), U.S. National Academies of Science, Engineering and Medicine (NASEM), the European Academies Science Advisory Council (EASAC) and the German National Academy of Sciences. Authors highlighted hijacking the human microbiome, immune system or genome and illegal cosmetic changes as future security implications. For example, enhancement of individual’s capabilities by off-label use of somatic editing (for muscle mass, neurology), e.g., for military purposes; germline modification of future populations. |
| *Kirkpatrick et al. (2018)* | USA | Other – Policy (Smith Richardson Foundation) | Speculative - Expert workshop with 14 different subject-matter experts including security, the life sciences, policy, industry, and, ethics in a two-year long study | CRISPR and Genome editing-related technology | Exploitation of CRISPR & gene drives | Biohacking, illegal gene editing and “neuro-hacking” | • Biohacker (outsider)  • Non-state actors (outsider)  • State actors (insider)  • Careless employee (insider)  • Rogue elements in a diagnostic lab (insider) | Consulted 14 different subject-matter experts including security, the life sciences, policy, industry, and, ethics in a two-year long study. Scenarios generated by the experts included dual-use / biosafety concerns. Examples included reckless actors exploiting CRISPR, modifying microbes to be more dangerous, hijacking the human microbiome, weaponizing gene therapies and releasing gene drives as weapons that could pose novel security risks for entomological warfare, agro-sabotage and ecocide. |
| *Hauptman et al. (2013)* | Israel | Other – Futures (Foresight) | Speculative - Horizon Scanning and Delphi methodology with 280 experts from academia and research institutes, 50% with security experience | Synthetic biology | “Genetic blackmail” &  exploited Synthetic biology technologies | “Genetic blackmail” | • Biohacker (outsider)  • Non-state actors (outsider) | 280 experts estimated the maturity of various emerging technologies, ranking each of 33 technologies by the likelihood they will pose a security threat, the easiness of malicious use and the societal spheres that would be most affected. Synthetic biology ranked as the sixth most mature technology by its potential for abuse. Also conducted a scenario workshop with selected technologies that may pose threats to security when combining “weak signal” technologies to “wild card” technologies (low probability, high impact). For example, ‘‘genetic blackmail’’ by misusing DNA of human individuals for extortion. |
| *Wintle et al. (2017)* | U.K. | Life Sciences (eLife) | Speculative - Horizon Scanning, Delphi and Scenario building with 27 participants from diversified backgrounds; natural sciences, engineering, social sciences,  and humanities | Gene drives, human genome editing, synthetic biology, microbiome- based therapies | Intended misuse of Synthetic biology | “Cyber-biocrime”, DIY drugs, biohacking, illegal gene editing and “Genetic blackmail” | • Hacker (outsider) | Ranked the top-scoring 20 issues from an initial list of 70 items grouped by how soon the issue could generate risks to society. Synthetic biology was estimated to be a near future (5 – 10 years) threat in the form of microbiome-therapies using engineered organisms. Reported the possibility of producing genetically engineered bacterial strains or viruses that could be introduced to the host microbiome using cell-based therapies; a synthetic biology technology originally used to prevent infection, that may be used to cause infection. |
| *Bress (2017)* | USA | Other - Naval studies (American Naval postgraduate school master’s dissertation) | Speculative - Scenario building and four global megatrends (globalization, urbanization, Internet of things/hyper-connected society, and  exponential technological growth) ranked with a three-point Likert scale (likely, possible, radical | Synthetic biology | Illegal use and manufacturing of drugs using emerging technology | DIY drug manufacturing and biohacking | • End user (outsider)  • Non-state actors (outsider)  • State actors (insider)  • Biohacker (outsider) | Forecast nefarious activity in the form of illegal use and manufacturing of synthetic “Do-It-Yourself” (DIY)-made or commercially available drugs tailored to genetic makeup of customer. Discussed development of non-biochemical drugs and their anticipated development by biohackers, possibly using neural interface technology for illicit synthetic drug use. |
| *DiEuliis et al. (2017)* | USA | Life Sciences (Health security) | Speculative - Author Speculation from a Senior Research Fellow at the Center for the Study of Weapons of Mass Destruction for the U.S. National Defense and a Professor of the University, Washington at the Departments of Neurology and Biochemistry, specialising in  Neuro-ethics | CRISPR | “Neuro-hacking” | “Neuro-hacking” | • ‘‘Do-It-Yourself’’ (DIY) community (outsider)  • Biohacker (outsider)  • State actors (insider)  • iGEM students (insider)  • Careless employee (insider)  • Rogue elements in a diagnostic lab (insider) | Collaborators including the Centre for the Study of Weapons of Mass Destruction conducted a narrative threat assessment of CRISPR on neural systems. Discussed “neuro-hacking” risks involving the indirect control of the brain through manipulation of the microbiome. |
| *Ali et al. (2016)* | USA and Abu Dhabi | Computer Science (IEEE Computer Society) | Speculative - Author Speculation from technical authors in Electrical and  Computer Engineering, hardware security and digital microfluidics | Digital Microfluidic Biochips (DMFB) | Supply chain attacks | “Cyber-biocrime” | • Rogue elements in a diagnostic lab (insider)  • End user (outsider) | DMFB device disruption / outcome modification with serious consequences for laboratory analysis, healthcare, and biotechnology innovation. Included both supply chain attacks (on manufacturing processes) and “Trojan”-like attacks, malware that works by misleading users of its true intent. |
| *Peccoud et al. (2018)* | USA | Life Sciences (Trends in Biotechnology – Cell Press review) | Speculative - Literature  Review | Biological systems integrated on digital systems (e.g. biomanufacturing processes) | Exploitation of the integrated systems between biology and the cyberspace - “Cyber-biocrime” | “Cyber-biocrime” | • Rogue elements in a diagnostic lab (insider)  • Nefarious actor (outsider) | Cyber-physical penetration of biotechnology workflows to:   - Produce toxic products by corrupting bioinformatics databases - Produce nefarious products by intercepting shipments / electronic orders - Compromising the operation of a vital biomanufacturing facility by disrupting computer-controlled processes (eg. uncoupling the actual parameters of a process from the data reported) |
| *Qu (2018)* | USA | Computer Science (Tufts school - undergraduate dissertation) | Speculative - Literature  Review | Genomic data | Genetic discrimination and/or blackmail through data breaches | Bio-discrimination | • Hacker (outsider) | Proposed cyberattack techniques that included ‘Sniffing’ - the theft/interception of data on network by capturing traffic, ‘Man-in-the-middle’ - eavesdropping in data transfer or transactions, ‘Buffer overflow’ - a software exploit that overruns an allocated memory block and leakage of unencrypted sensitive data, and cloud computing exploits that attack vulnerabilities in internet protocol, data recovery and access to management interfaces. |
