# Supplementary material for: A Systematic Review of the Criminogenic Potential of Synthetic Biology and Routes to Future Crime Prevention
Source: Front Bioeng Biotechnol. 2020 Oct 6;8:571672. doi: 10.3389/fbioe.2020.571672 (PMC7573185; doi:10.3389/fbioe.2020.571672)
Supplement: Supplementary file 2 [file Table_2.docx]

Supplementary Material

**Supplementary Table 2. Identified factors shaping future biocrime.** Identified factors extracted from the 15 reviewed studies that promote the occurrence of the eight identified crime harvests.

| Societal feature | Government | Technology | Industry | Public/user | Culture |
| --- | --- | --- | --- | --- | --- |
| Identified factors | Insufficient national regulation  (Fears & Meulen, 2017) | Hyper-connectivity and devices  (Bress, 2017) | Increasing numbers of commercial service providers  (Ali et al., 2016; Dieuliis & Giordano, 2017; Kirkpatrick J, Koblentz GD, Palmer MJ, Perello E, Relman DA, 2018; Ney P, Koscher K, Organick L, Ceze L, 2017; Qu, 2019; Wintle BC, Boehm CR, Rhodes C, Molloy JC, Millett P, Adam L, 2017) | Public literacy increasing  (Ali et al., 2016) | Open source culture  (Backes et al., 2016; Fears & Meulen, 2017; Franzosa EA, Huang K, Meadow JF, Gevers D, Lemon KP, Bohannan BJ, 2015; Wintle BC, Boehm CR, Rhodes C, Molloy JC, Millett P, Adam L, 2017) |
|  | Increasing defense agency research in biological engineering  (Wintle BC, Boehm CR, Rhodes C, Molloy JC, Millett P, Adam L, 2017) | Hyper-personalization of health and malign biohacking  (Bress, 2017) | "Democratization of Biotechnology" e.g. community labs  (Kirkpatrick J, Koblentz GD, Palmer MJ, Perello E, Relman DA, 2018) | Insufficient public confidence  (Fears & Meulen, 2017) |  |
|  | Insufficient international legislation  (Kirkpatrick J, Koblentz GD, Palmer MJ, Perello E, Relman DA, 2018) | Cloud computing  (Hauptman & Sharan, 2013; Ney P, Koscher K, Organick L, Ceze L, 2017; Qu, 2019) | Shifting ownership models in biotechnology and rise of start-ups  (Ali et al., 2016; Kirkpatrick J, Koblentz GD, Palmer MJ, Perello E, Relman DA, 2018; Qu, 2019; Wintle BC, Boehm CR, Rhodes C, Molloy JC, Millett P, Adam L, 2017) | Increased capability and number of users  (Ali et al., 2016; Kirkpatrick J, Koblentz GD, Palmer MJ, Perello E, Relman DA, 2018) |  |
|  | Preparing for a bioeconomy  (Wintle BC, Boehm CR, Rhodes C, Molloy JC, Millett P, Adam L, 2017) | Decreasing cost of DNA synthesis  (Dieuliis & Giordano, 2017; Fears & Meulen, 2017; Kirkpatrick J, Koblentz GD, Palmer MJ, Perello E, Relman DA, 2018; Ney P, Koscher K, Organick L, Ceze L, 2017; Ney PM, Organick L, Koscher K, Kohno T, 2018; Qu, 2019) |  | Increased activity of end user  (Ali et al., 2016) |  |
|  |  | Increasing availability of inexpensive tools  (Dieuliis & Giordano, 2017) |  |  |  |
|  |  | New technologies and new data |  |  |  |
|  |  | Increasing levels of data produced, stored and handled online  (Ayday E, Raisaro JL, McLaren PJ, Fellay J, 2013; Backes et al., 2016; Franzosa EA, Huang K, Meadow JF, Gevers D, Lemon KP, Bohannan BJ, 2015; Qu, 2019) |  |  |  |
|  |  | Increasing DNA sequencing speeds  (Ney P, Koscher K, Organick L, Ceze L, 2017; Ney PM, Organick L, Koscher K, Kohno T, 2018) |  |  |  |
|  |  | Increasing cyber-physical interfaces  (Ayday E, Raisaro JL, McLaren PJ, Fellay J, 2013; Faezi S, Chhetri SR, Malawade AV, Chaput JC, Grover WH, Brisk P, 2019; Ney P, Koscher K, Organick L, Ceze L, 2017; Peccoud J, Gallegos JE, Murch R, Buchholz WG, 2018; Wintle BC, Boehm CR, Rhodes C, Molloy JC, Millett P, Adam L, 2017)) |  |  |  |
|  |  | Low levels of cyberbiosecurity hygiene  (Faezi S, Chhetri SR, Malawade AV, Chaput JC, Grover WH, Brisk P, 2019; Ney P, Koscher K, Organick L, Ceze L, 2017; Peccoud J, Gallegos JE, Murch R, Buchholz WG, 2018) |  |  |  |
|  |  | Prevalence of automated cyber-physical systems  (Faezi S, Chhetri SR, Malawade AV, Chaput JC, Grover WH, Brisk P, 2019; Peccoud J, Gallegos JE, Murch R, Buchholz WG, 2018; Wintle BC, Boehm CR, Rhodes C, Molloy JC, Millett P, Adam L, 2017) |  |  |  |
